# A Preliminary Study of the Effects of SurAsleep on Relieving Symptoms of Sleep Disorders

**Published:** 2015-06

**Authors:** Qingwen Xie, Tian Zhou, Lawrence Yen, Andrew Song, Mina Shariff, Tuong Nguyen, Jianyu Rao, Rong Shi

**Affiliations:** 1 Department of Preventive Medicine, School of Public Health, Shanghai Jiao Tong University School of Medicine, Shanghai 200001, China;; 2 Department of Pathology and Laboratory Medicine, David Geffen School of Medicine, University of California at Los Angeles, CA 90095, USA;; 3 Department of Research, DRM Resources, 1683 Sunflower Avenue, Costa Mesa, CA 92626, USA

**Keywords:** PSQI, sleep disorder, SurAsleep, self-assessment, Questionnaire, sleep quality, somnipathy

## Abstract

Sleep disorder is a common condition in the general population. Conventional sedative-hypnotic drug therapy may not be appropriate for many patients suffering from only mild-to-moderate sleep disorders. SurAsleep, a nutritional supplement that has been used by patients with sleep disorders in the United States, shows promising effects in improving sleep disorders and enhancing sleep quality. However, double-blinded, randomized and controlled studies have not been performed to determine the efficacy of the supplement. We conducted this study on individuals suffering from mild-to-moderate sleep disorders in Shanghai, China. In this study, we randomly assigned 100 participants over the age of 50 years old with symptoms of sleep disorders to a 12-week treatment with either SurAsleep or placebo. The results were measured by a self-administrated questionnaire on changes in symptoms, which covered 3 phases of sleep: the falling-asleep stage, the sleeping stage and the waking-up stage. We also measured the changes after the 12-week intervention using the Pittsburgh Sleep Quality Index (PSQI), including 7 dimensions of sleeping. All symptoms, dimension-specific PSQI scores and total PSQI scores showed significant improvement after using SurAsleep. In this study, SurAsleep has shown potent effects in relieving somnipathy-related symptoms and improving sleep quality in sleep disorder patients.

## INTRODUCTION

Sleep disorder, or somnipathy, is a disorder concerning sleep patterns of an individual. Mainly manifested as insomnia (1), somnipathy is associated with undesirable physiological or mental conditions, and influenced by many factors (disease, environment, psychology, mentality, society, etc.) (2, 3). Some sleep disorders are serious enough to interfere with normal physical, mental and emotional functioning (4), negatively affecting the quality of life, especially if it remains a chronic condition.

In recent years, somnipathy has become a common physiological and psychological disorder in many regions around the world. Several investigations demonstrated that 3.5% to 30% of adults suffer from somnipathy (5-7). Many patients suffering from somnipathy use sedative-hypnotic drugs to alleviate the symptoms. These drugs cause various side effects such as dizziness, drowsiness, memory damage, cognitive impairment and dependence, and withdrawal symptoms such as anxiety (8-10). These drugs are inappropriate for most patients suffering from only mild-to-moderate sleep disorders. For these patients, the main disease manifestations are difficulty in falling asleep, early awakening, restless sleep, difficulty in continuing sleep after waking, dreaminess, discomfort after waking up and daytime fatigue. Therefore, natural products or nutritional supplements with perceivably less side effects may be useful for such a patient population.

SurAsleep is a nutritional supplement that promotes deep, restful sleep. The main chemical ingredients include calcium, magnesium, valerian root extract (*Valeriana officinalis*), oat straw extract (*Avena sativa*), L-theanine and melatonin.

On “World Sleep Day” in 2002, a survey conducted by the International Foundation for Mental Health and Neurosciences (IFMHN) declared that 45.5% of the population in China suffered from insomnia (11). Insomnia or sleep disorders are highly common in Chinese populations (12). In order to evaluate the sleep-enhancing effects of SurAsleep, we conducted this double-blinded, randomized, controlled study on SurAsleep in Shanghai, China, to evaluate its effects through the changes in symptoms and improvements in sleeping quality.

The primary objective of the study was to determine whether the daily use of SurAsleep could relieve clinical symptoms of human subjects suffering from insomnia. The secondary objective was to measure the changes in PSQI before and after the intervention, including dimension-specific scores and total score.

## METHODS

### Participants

The study took place from November 2011 to February 2012. The Pittsburgh Sleep Quality Index (PSQI) (13) was applied as recruitment criterion. PSQI is a standard measuring scale assessing sleep quality through 7 dimensions, including subjective sleep quality, time of falling asleep, sleeping time, sleep efficiency, somnipathy, hypnotic drugs and diurnal dysfunction, based on the sleep quality in the past month. Each specific item is assigned a score from 0 to 3 points. The cumulative score totaling all 7 dimensions that was used to index sleep quality ranged from 0 to 21, with a higher score indicating poorer sleep quality. One hundred participants (37 males and 63 females) between the ages of 50 and 89 years old (mean age 61.17), who suffered from mild-to-moderate sleep disorders, were recruited from the Lujiazui community health service center in Shanghai, China. Cases suffering from severe health conditions or complications, such as tumors, cardiac diseases and other serious diseases, and candidates who had used sleeping pills or sleep-enhancing health products within 2 weeks of the study were excluded. All participants provided written, informed consent. The recruitment period was 2 weeks, washout period was 1 week and study (follow-up) period was 12 weeks.

### Technical information

All of the 100 eligible participants were randomly assigned into either the SurAsleep group or the placebo group with 50 patients in each group. The randomization was performed using a predetermined randomization code which was generated by a random number generator. The numbers generated were placed in sealed envelopes, and a serial number was assigned to each envelope according to the sequence of allocation of the randomized number. Each envelope was then opened sequentially according to the admission sequence of subjects. Subjects as well as physicians and nurses were blinded to the patients’ allocation. The participants received similar-looking capsules differentiated by color-coded bottles (white bottles for SurAsleep and yellow ones for placebo), and both study subjects and investigators, including the study Principal Investigator (Shi, Rong), did not know the specific color code until after the study was completed.

Both the SurAsleep capsules and the placebo were manufactured by Robinson Pharma (Costa Mesa, California, USA). The ingredients of the SurAsleep capsules included calcium, magnesium, valerian root extract (*Valeriana officinalis*), oat straw extract (*Avena sativa*), theanine and melatonin, while the placebo was mainly composed of flour. Each participant was instructed to take 1 capsule 30 to 60 minutes before bedtime. The treatment lasted for 12 weeks and medications were dispensed every month during follow-up sessions. The total follow-up period for every participant lasted for 3 months.

The experimental results were measured by a self-administrated questionnaire on changes in symptoms and compared between the 2 groups. Symptoms of interest covered 3 phases of sleep: the falling-asleep stage (level of difficulty in falling asleep), the sleeping stage (ease of waking up, dreaminess), and the waking-up stage (occurrence of early awakening or diurnal fatigue). Each symptom was graded from 0 to 5 (0 indicating no such symptom exists and 5 indicating a severe degree of the symptom) by the participant.

According to the PSQI scoring standard, we classified subjects as somnipathy cases if the PSQI total score exceeded 5 points or non-somnipathy if PSQI total score was equal to or less than 5 points. Somnipathy-related symptoms and PSQI questionnaires were conducted at baseline and the end of intervention for every eligible participant. Self-reported side effects and adherence were recorded along with follow-up.

### Statistical analysis

Baseline variables were compared between the 2 groups with Student’s t-test for continuous variables and Chi-square test or Fisher’s exact test for nominal variables. Paired t-test was used to measure changes before and after the intervention besides the above methods. The statistical package we used for data analysis was Stata/MP 11.2. The statistically significant alpha level we chose was 0.05. All analyses were applied by intent-to-treat (ITT) analysis. All *P*-values were 2-sided.

## RESULTS

Of the 100 eligible participants who underwent randomization, 47 were assigned to the SurAsleep group and 53 to the placebo group, with no loss to follow-up until the end of the study. There was no significant difference in adherence between the 2 groups, and no significant adverse effects reported until the end of the study. The baseline demographic characteristics were compatible (Table 1). Similarly, the somnipathy-related symptoms (difficulty in falling asleep; early awakening; ease of waking up; difficulty sleeping after waking up; dreaminess; discomfort after waking up; diurnal fatigue), PSQI scores (duration of sleep; sleep disturbance; sleep latency; day dysfunction due to sleepiness; sleep efficiency; overall sleep quality; need meds to sleep) and the proportions of participants with PSQI total score > 5 points in each group were also compatible at baseline (Table 1).

**Table 1:** Baseline Characteristics of the two groups

Characteristics	SurAsleep (N=47)	Placebo (N=53)	Total (N=100)

Gender - no.			
Male	18	19	37
Female	29	34	63
Age - year^a^	60.09 ± 7.69	62.13 ± 6.83	61.17 ± 7.28
Had chronic disease history - no. (%)	17 (36.17%)	13 (24.53%)	
Had alcohol intake history - no. (%)	10 (21.28%)	10 (18.87%)	
Had used drugs to help sleep recently - no. (%)	11 (23.40%)	13 (24.53%)	* *
Course of using hypnotic drugs – month	12.55 ± 12.60	20.46 ± 11.87	
**Somnipathy-Related Symptoms** b			
Difficulty in falling asleep	3.26 ± 1.15	3.23 ± 1.09	
Early awakening	3.11 ± 1.01	3.30 ± 0.99	
Ease of waking up	3.13 ± 0.99	2.96 ± 1.00	
Difficulty sleeping after waking up	2.98 ± 1.09	3.00 ± 1.04	
Dreaminess	2.74 ± 1.13	2.49 ± 1.05	
Discomfort after waking up	2.34 ± 1.09	2.08 ± 0.98	
Diurnal fatigue	2.40 ± 0.95	2.42 ± 0.99	
**PSQI Scores** c			
Duration of sleep	1.15 ± 1.042	1.25 ± 1.108	
Sleep disturbance	1.49 ± 0.505	1.42 ± 0.570	
Sleep latency	2.38 ± 0.768	2.32 ± 0.850	
Day dysfunction due to sleepiness	0.94 ± 0.704	0.89 ± 0.670	
Sleep efficiency	1.15 ± 1.197	1.40 ± 1.214	
Overall sleep quality	1.83 ± 0.481	1.87 ± 0.652	
Need meds to sleep	0.70 ± 0.998	0.70 ± 1.137	
**Total** d	**9.64 ± 3.541**	**9.83 ± 4.214**	
**Proportion of participants whose PSQI total score > 5 points** e	**95.74%**	**83.02%**	

a Plus–minus values were means ± SD, the same hereinafter;

b Scores on the somnipathy-related symptoms can range from 0 (no symptoms) to 5 (severe symptoms);

c Scores on the PSQI dimensional scores can range from 0 to 3. Higher score indicates poorer sleep quality in this dimension;

d PSQI total score can range from 0 to 21. Higher score indicates poorer sleep quality;

e PSQI Total score ≤ 5 associated with good sleep quality; Total > 5 associated with poor sleep quality.

After the 12-week intervention, profound differences were observed between the two groups (Table 2). All the symptom scores (difficulty in falling asleep; early awakening; ease of waking up; difficulty sleeping after waking up; dreaminess; discomfort after waking up; diurnal fatigue), scores of the 7 dimensions of PSQI index (duration of sleep; sleep disturbance; sleep latency; day dysfunction due to sleepiness; sleep efficiency; overall sleep quality; need meds to sleep) and the total PSQI score in the SurAsleep group were significantly reduced after the intervention (Table 3).

**Table 2:** Symptom scores and PSQI scores after intervention

	SurAsleep	Placebo	*P*-value^f^

**Somnipathy-Related Symptoms** b			
Difficulty in falling asleep	2.11 ± 0.814^a^	3.13 ± 1.127	<0.001
Early awakening	2.28 ± 0.852	3.26 ± 0.944	<0.001
Ease of waking up	2.21 ± 0.832	2.83 ± 0.995	0.001
Difficulty sleeping after waking up	2.28 ± 0.852	2.94 ± 0.969	<0.001
Dreaminess	1.94 ± 0.818	2.40 ± 0.968	0.012
Discomfort after waking up	1.57 ± 0.683	2.04 ± 0.898	0.005
Diurnal fatigue	1.66 ± 0.760	2.36 ± 0.963	<0.001
**PSQI dimensions** c			
Duration of sleep	0.34 ± 0.700	0.92 ± 0.997	0.001
Sleep disturbance	1.09 ± 0.282	1.42 ± 0.497	<0.001
Sleep latency	1.43 ± 0.683	2.30 ± 0.868	<0.001
Day dysfunction due to sleepiness	0.40 ± 0.496	0.91 ± 0.687	<0.001
Sleep efficiency	0.36 ± 0.792	1.02 ± 1.185	0.002
Overall sleep quality	0.74 ± 0.607	1.79 ± 0.689	<0.001
Need meds to sleep	0.15 ± 0.416	0.66 ± 1.192	0.006
**Total** d	**4.51 ± 2.645**	**9.02 ± 4.088**	<0.001
**Proportion of participants whose PSQI total score > 5 points** e	**19.15%**	**73.58%**	<0.001

a Plus–minus values were means ±SD, the same hereinafter;

b Scores on the somnipathy-related symptoms can range from 0 (no symptoms) to 5 (severe symptoms);

c Scores on the PSQI dimensional scores can range from 0 to 3. Higher score indicates poorer sleep quality in this dimension;

d PSQI total score can range from 0 to 21. Higher score indicates poorer sleep quality;

e PSQI Total score ≤ 5 associated with good sleep quality; Total > 5 associated with poor sleep quality. The *P*-value was based on chi-square test;

f Based on t-test except “Proportion of participants whose PSQI total score > 5 points”.

**Table 3:** Paired t-test for symptom scores and PSQI scores before and after the intervention

	SurAsleep		Placebo	
	Mean Difference	*P*-value	Mean Difference	*P*-value

**Somnipathy-Related Symptoms** a				
Difficulty in falling asleep	1.15	<0.001	0.09	0.023
Early awakening	0.83	<0.001	0.04	0.622
Ease of waking up	0.91	<0.001	0.13	0.018
Difficulty sleeping after waking up	0.70	<0.001	0.06	0.182
Dreaminess	0.81	<0.001	0.09	0.168
Discomfort after Waking up	0.77	<0.001	0.04	0.485
Diurnal fatigue	0.74	<0.001	0.06	0.411
**PSQI dimensions** b				
Duration of sleep	0.81	<0.001	0.32	<0.001
Sleep disturbance	0.40	<0.001	0	1.000
Sleep latency	0.96	<0.001	0.02	0.659
Day dysfunction due to sleepiness	0.53	<0.001	-0.02	0.569
Sleep efficiency	0.79	<0.001	0.38	<0.001
Overall sleep quality	1.09	<0.001	0.08	0.159
Need meds to sleep	0.55	<0.001	0.04	0.742
**Total** c	**5.13**	**<0.001**	**0.81**	**<0.001**

a Original scores before paired t-test on the somnipathy-related symptoms can range from 0 (no symptoms) to 5 (severe symptoms);

b Original scores before paired t-test on the PSQI dimensional scores can range from 0 to 3. Higher score indicates poorer sleep quality in this dimension;

c Original scores before paired t-test PSQI total score can range from 0 to 21. Higher score indicates poorer sleep quality.

The total PSQI score was markedly decreased more than half, relative to the placebo group, while for the placebo group only a slight decrease was observed (Table 1 and Table 3). Furthermore, the proportion of SurAsleep group participants with poor sleep quality, indicated by PSQI total score > 5 points, decreased sharply from 95.74% to 19.15%, while in the placebo group this rate decreased only from 83.02% to 73.58% (Table 1 and Table 2).

## DISCUSSION

In this randomized preliminary study, the 2 groups were well balanced for baseline-demographics and severity of insomnia-related symptoms. After the 12-week intervention, SurAsleep showed remarkable effects, at least in the short-term, in alleviating sleep disorder symptoms relative to the placebo. In our study, participants using SurAsleep for 3 months showed significant improvements in most symptom scores compared to the placebo group and no significant adverse effects were reported.

SurAsleep also displayed improvements in PSQI index assessment. All 7 dimensions and the total scores improved significantly. Using the total score of 5 as the cutoff point for overall sleep quality, we found in the SurAsleep group, subjects with overall score > 5 decreased from 95.74% at the baseline to 19.15% after intervention, whereas the placebo group only showed a slight decrease (from 83.02% to 73.58%), which may be due to the placebo effect.

SurAsleep is a nutritional supplement that has been marketed in the U.S. for many years. Its observed properties may be due to its main chemical ingredients, which include calcium, magnesium, valerian root extract (*Valeriana officinalis*), oat straw extract (*Avena sativa*), theanine and melatonin. Although our study could not determine which specific ingredient(s) was responsible for the observed effect, there are several possible modes of action that might have been accountable for producing our recorded results. For example, SurAsleep contains melatonin, which has been shown to produce significant reductions in sleep onset latency, and improved sleep quality and morning alertness in insomnia patients over the age of 55 (14, 15). SurAsleep’s effects could therefore be due to either the component of melatonin working alone or the synergistic effect of this ingredient working together with calcium, magnesium, etc., which were selected based on their individual benefits to overall sleep quality.

Calcium is an important neurotransmitter (16). According to the manufacturer, it may have effects in strengthening the inhibition process in the cerebral cortex, and adjusts the balance between excitement and inhibition. Studies have shown that lack of calcium may affect the normal metabolism of neurons in the brain and causes prolonged excitement of the cerebral cortex resulting in sleep difficulties (17, 18). Low levels of magnesium are associated with the reduced capability of stress management. In practice, magnesium and calcium may be administered simultaneously as a natural tranquilizer. Valerian itself does not cause syngignoscism, however, it can enhance the effect of benzodiazepine (19). Oats is a natural food product rich in protein and other nutrients. The husk of oats is beneficial to improving levels of serotonin, which may help to improve sleep quality and maintain sedation (20). Theanine, extracted from green tea, may affect the secretion of neurotransmitters in the brain (21), antagonize caffeine-caused excitation and relieve psychological tension (22). Melatonin is an endocrine hormone, secreted by the pineal gland that regulates circadian rhythms and sleep by specific receptors (23). No noticeable side effects were observed during our study, but SurAsleep contains calcium and magnesium, which might be potentially harmful in patients with high baseline calcium and/or magnesium.

## CONCLUSION

In this double-blinded, randomized, preliminary, short-term study, SurAsleep showed beneficial effects in improving sleep quality, including all 3 sleep stages, in individuals suffering from sleep disorders. Further larger scale clinical studies may be warranted to validate our observations.

## ABBREVIATIONS

PSQI: Pittsburgh Sleep Quality Index
IFMHN: International Foundation for Mental Health and Neurosciences
ITT: intent-to-treat
